# Trauma: Harmful effect of diagnostic labeling and iatrogenic intervention for the recovery process

**DOI:** 10.1192/j.eurpsy.2021.1919

**Published:** 2021-08-13

**Authors:** C. Martín Villarroel, L. Carpio Garcia, J. Matsuura, M. Sánchez Revuelta, G. Belmonte García, J. Dominguez Cutanda, M. Fernández-Torija Daza, E. García

**Affiliations:** Psiquiatría, Complejo Hospitalario Universitario de Toledo, Toledo, Spain

**Keywords:** traumatic factors, dissociation, excision mechanism, evolutionary development

## Abstract

**Introduction:**

We know the coexistence of traumatic factors (loss of affective relationships, experiences of abuse, extreme risk situations, etc.) is common in psychiatric pathologies in which level of stress experienced exceeds normal capacity of the person, favoring the appearance of dissociative or excision mechanisms. A common mistake is to pathologize them and try to eliminate them.

**Objectives:**

The objective of this paper is to study trauma and defense mechanisms involved, in order to carry out a better approach.

**Methods:**

A bibliographic search was performed from different database (Pubmed, TripDatabase) about trauma, mechanisms involved and the construction of identity.

**Results:**

We know neural pathways mature asymmetrically in evolutionary development (functions related to attention, concentration and executive function having special importance) and thus, traumas occurred in moments of greatest vulnerability such as early childhood, can damage and interfere with the correct integration of neural processes, producing disproportionate and unnecessarily maintained alert responses (common basis for many pathologies such as borderline personality disorder or traumatic psychosis). In response to this, reactive mechanisms are produced (such as dissociation or cleavage) that are not necessarily pathological and therefore, we should not always intervene by eliminating them because they often function as a protective factor, allowing to preserve functioning and favoring recovery.

**Conclusions:**

In conclusion, we need a better understanding of mechanisms involved in trauma, executive function and the alarm system beyond anxiety reactions, trying to understand the function of symptom without eliminating it, but evaluating whether there are healthier alternatives can be promoted for the complete recovery of the patient.

**Disclosure:**

No significant relationships.

